# In Situ Adsorption of Red Onion (*Allium cepa*) Natural Dye on Cellulose Model Films and Fabrics Exploiting Chitosan as a Natural Mordant

**DOI:** 10.1021/acsomega.2c06650

**Published:** 2023-02-01

**Authors:** Rafael Grande, Riikka Räisänen, Jinze Dou, Satu Rajala, Kiia Malinen, Paula A. Nousiainen, Monika Österberg

**Affiliations:** † Department of Bioproducts and Biosystems, School of Chemical Engineering, Aalto University, Vuorimiehentie 1, 02150Espoo, Finland; ‡ Craft Science, University of Helsinki, Siltavuorenpenger 10, 00014Helsinki, Finland

## Abstract

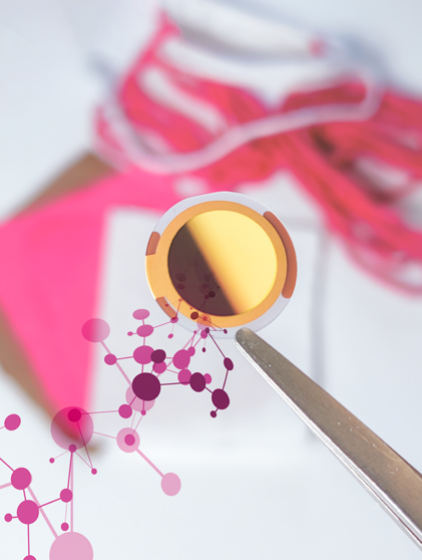

Synthetic dyes and chemicals create an enormous impact on environmental pollution both in textile manufacturing and after the product’s lifetime. Biobased plant-derived colorants and mordants have great potential for the development of more sustainable textile dyeing processes. Colorants isolated from biomass residues are renewable, biodegradable, and usually less harmful than their synthetic counterparts. Interestingly, they may also bring additional functions to the materials. However, the extraction and purification of the biocolorants from biomass as well as their dyeing efficiency and color fastness properties require a more thorough examination. Here, we extracted red onion (*Allium cepa*) skins to obtain polyphenolic flavonoids and anthocyanins as biocolorants, characterized the chemical composition of the mixture, and used a quartz crystal microbalance and thin films of cellulose nanofibrils to study the adsorption kinetics of dyes onto cellulose substrates in situ. The effect of different mordants on the adsorption behavior was also investigated. Comparison of these results with conventional dyeing experiments of textiles enabled us to determine the interaction mechanism of the dyes with substrates and mordants. Chitosan showed high potential as a biobased mordant based both on its ability to facilitate fast adsorption of polyphenols to cellulose and its ability to retain the purple color of the red onion dye (ROD) in comparison to the metal mordants FeSO_4_ and alum. The ROD also showed excellent UV-shielding efficiency at low concentrations, suggesting that biocolorants, due to their more complex composition compared to synthetic ones, can have multiple actions in addition to providing aesthetics.

## Introduction

Natural dyes have been used for centuries to add value, individuality, and even social status to textiles. In the Roman Empire, for example, only the aristocracy and eventually emperor Gaius Julius Caesar were allowed to wear a rare purple dye obtained from sea snails. This precious dye was one of the most expensive and strived trade goods in history.^1^ The tradition of natural dyeing nearly disappeared after the development of synthetic dyes in the mid-19th century. Synthetic dyes replaced natural ones in the modern textile finishing industry as they were cheap and easy to produce and apply. However, current aims toward biobased products and sustainable processes have changed the picture. Most synthetic dyes are derived from petrochemical sources, and their production may require hazardous chemicals posing environmental challenges. The textile industry produces yearly over 200 000 tons of harmful substances released into effluents.^2^ These pollutive processes have rekindled academic and commercial interest in natural dyes to bring the textile industry up to date with modern environmental and sustainability standards.

Sustainable textile production can benefit from natural (and biobased) dyes because they are of renewable origin, biodegradable, and often less harmful compared to synthetic ones.^3^ However, the efficiency of their extraction and purification needs to be considered in detail as well as how to firmly attach them to the fabrics in a sustainable manner. In a recent study, different extraction and purification methods of natural dyes were compared, and the results suggest that the final form of liquid dye was the most feasible, as the drying phase increases energy consumption considerably.^4^ EU legislation recommends liquid-type colorants whenever convenient to use to decrease the health hazards caused by the dusting of powder-form dye.^5^

Aside from color, the extracts derived from natural sources have also been reported as attractive alternatives to add functionality to materials in a variety of applications, including medicinal products,^6^ coatings,^7^ and smart packaging.^8−10^ The multifunctional aspect of biobased dyes relies on the structural diversity of bioactive compounds found in their extracts (i.e., curcuminoids, quinones, tannins, flavonoids, alkaloids).

Natural colorants are typically derived from plants and minerals,^11^ but their extraction from agricultural biowaste and other industrial byproducts provides an opportunity to add value to biomass, thus supporting the circular economy.^4^ For instance, the crop production of onion (*Allium cepa*) generates more than half a million tons of biowaste in Europe alone.^11,12^ Red onion outer layers (skins), in particular, are rich in flavonoids, such as anthocyanidins and anthoxanthins, which are well known for their antimutagenic, antioxidant, UV-shielding as well as coloring properties.^13^

Natural dyes are generally not readily absorbed by textile fibers. The direct adsorption of natural dyes onto cellulosic fibers is even more unlikely because the cellulose surface becomes slightly negatively charged when soaked in water (at neutral pH), causing electrostatic repulsion between the fibers and the equally negatively charged dyes. In fact, anchoring agents known as mordants are often incorporated into the dyeing process to enhance dye uptake. Historically, polyphenols like tannic acids and tannins were first introduced as mordants. However, the most commonly used mordants are metal ions in their salt form, such as ferrous sulfate (FeSO_4_) and potassium aluminum sulfate (a.ka. Alum, KAl(SO_4_)_2_). The metal polyvalent cations form strong coordination complexes with the negatively charged organic ligands, i.e., the fibers and dye molecules, binding them effectively and resulting in a deeper color shade and improved color fastness.^14^ Despite their effectiveness, the use of metal mordants in biocolored textiles decreases the sustainability of using biocolorants because heavy metal ion contamination of wastewater causes the majority of environmental hazards in the textile industry. Hence, alternative, less polluting mordants are needed.

Biobased mordants are emerging as promising alternatives to metal mordants.^14−16^ Among them, chitosan, a renewable polyelectrolyte prepared via deacetylation of chitin that is available from crustacean wastes of the fishing industry, represents an interesting biomordant candidate owing to its high positive charge in acidic media. The primary amino groups in the chitosan backbone are easily protonated in mildly acidic conditions, which can lead to a strong electrostatic association with negatively charged fibers and electronegative groups in natural dyes. In addition to this unique characteristic, chitosan has been demonstrated to have many other functional properties, such as biocompatibility, and antimicrobial activity, that may have a synergistic effect with most natural dyes.^17−19^ Chitosan has been exploited as a textile finishing and sizing agent, but its application as a functional biomordant in natural dyeing has remained nearly unexplored.^19−25^ Moreover, the scarcity of literature addressing the more fundamental aspects of natural dyeing, such as dye fixation mechanisms, chemical characterization of dyes, and optimization of dyeing parameters, highlights the need for more systematic research to achieve a better understanding of dye adsorption dynamics as well as the influence of process parameters. Furthermore, clarifying the kinetics of dye adsorption on and desorption from cellulosic fibers is critical for the optimization of dyeing processes but remains to date nearly unexplored in scientific literature. The ultimate goal of gaining a better understanding of these issues is to make natural dyes and mordants an economically viable option.

**Figure 1:**
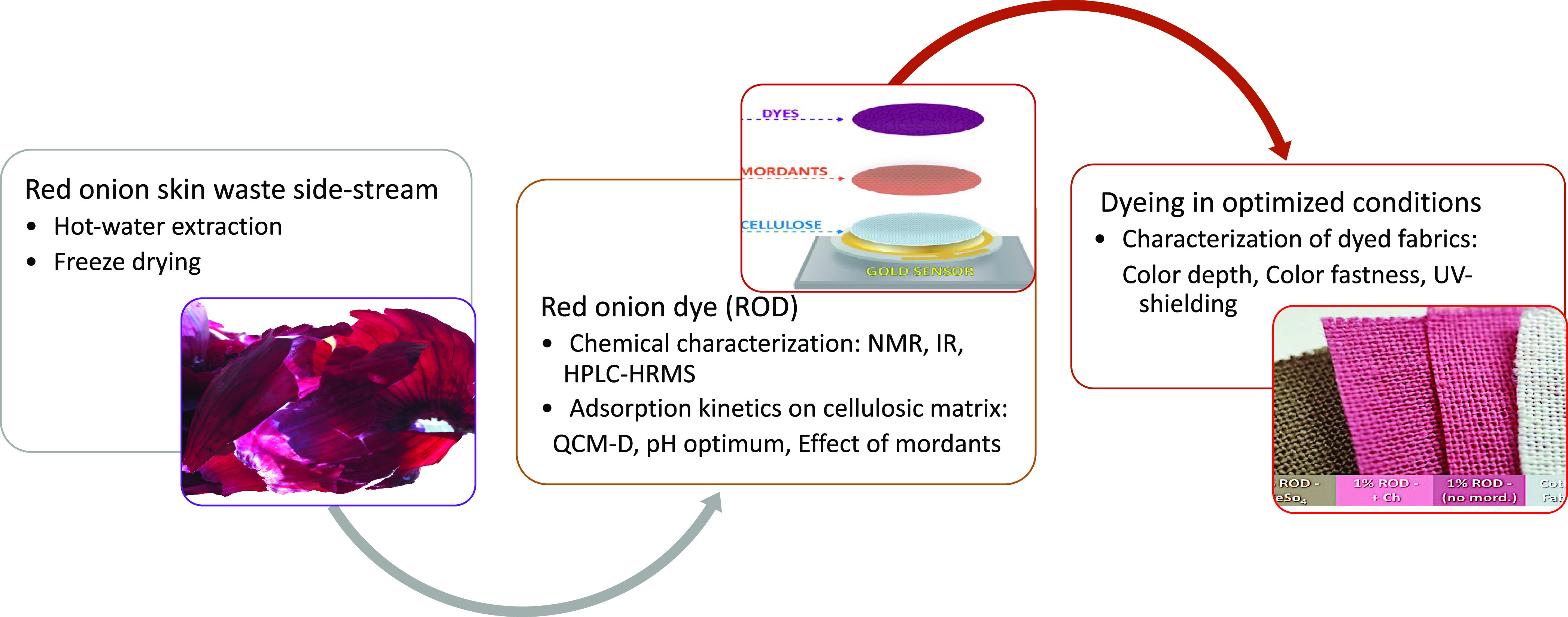
Scheme of the workflow on the dyeing method development of the red onion dye (ROD) extract on cellulosic fibers.

In this work, an analytical study was carried out to gain a more detailed understanding of the dyeing process on cellulosic materials. Quartz crystal microbalance with dissipation monitoring (QCM-D) was employed to detect the in situ adsorption in real time, allowing mordant and dye adsorption kinetics and adsorbed amounts to be determined. A natural dye obtained from red onion skins (*A. cepa*) and the efficiency of chitosan as a mordant was investigated. The adsorption kinetics and optical properties of CNF thin films dyed directly with the red onion dye (ROD) were compared to films mordanted with a conventional metal (FeSO_4_) or chitosan. In addition, the ROD was structurally and chemically characterized using nuclear magnetic resonance (NMR) spectroscopy, high-performance liquid chromatography combined with high-resolution mass spectrometry (HPLC-HRMS), and Fourier transform infrared spectroscopy (FTIR). The surface-sensitive dye adsorption studies were correlated to industrial scale simulating dyeing experiments of cotton fabrics with color-fastness testing. Furthermore, ROD’s potential as an effective UV-shielding agent for functional textiles was demonstrated by UV–visible (UV–Vis) spectrophotometry of cellulose-dyed films. The scheme of this work is shown in Figure 1.

## Experimental Section

### Materials

The red onion (*A. cepa*, cultivar Red Baron) was grown in Siilinjärvi, Finland, and obtained from Kesko (Helsinki, Finland). Onion skins were manually separated, dried, and stored with no further pretreatments carried out before experiments. The textile fabric used in dyeing was single-knit cotton (CO 95%, EA 5%) with a square mass of 205 g/m^2^ from Nanso (Helsinki, Finland). Distilled water was used in dyeing, and the following mordants: analytical grade FeSO_4_·7H_2_O (Sigma-Aldrich, Darmstadt, Germany), technical grade KAl(SO_4_)_2_·12 H_2_O (J. T. Baker, Deventer, The Netherlands), and chitosan (75–85% deacetylated, medium molecular weight, CAS 9012-76-4, Sigma-Aldrich).

Methanol and acetonitrile, used in HPLC spectroscopic measurements, were from Honeywell Riedel-de Haën (Charlotte, NC). Formic acid (≥99%) was from VWR International (Radnor, PA). D_2_O, DMSO-*d*_6_, pyridine-*d*_5_, 1,3,5-trioxane, arabinose, rhamnose, galactose, glucose, xylose, mannose, galacturonic acid, and quercetin were from Sigma-Aldrich, Finland.

The CNF suspension was prepared from never-dried hardwood birch from a Finnish pulp mill. To enhance fibrillation, the pulp fibers were first washed into the sodium form^26^ and then fibrillated by passing six times through the chamber of a fluidizer (Microfluidics, M-110Y, Microfluidics Int. Co., Newton, MA). The obtained CNF hydrogel typically exhibits a low charge (−4 mV measured ζ values) and fibril diameters around 10 nm.^27^ The CNF suspension was diluted to 1.0 g/L in deionized water and ultrasonicated at 25% amplitude for 5 min, using a Branson Sonifier S-450 D to further reduce the cellulose bundles. The diluted CNF suspension was then centrifuged at 7000*g* for 40 min, and the supernatant fraction with the finest CNF fibrils (about 0.15 wt % dry matter content) was collected and used for the preparation of CNF films.

### Dye Extraction

Red onion skins were added to a reactor in an onion skin-to-water mass ratio of 1:20. Water-soluble compounds were extracted with constant stirring (250 rpm) at 80 °C for 60 min. To remove the remaining insoluble particles, the extract was filtered twice (VWR filter paper with a 12–15 μm pore size) followed by centrifugation (4500*g*, 20 min). The filtrate was then freeze-dried and ground to obtain ROD in a yield of ca. 6 g/L. The purple powder was stored in a cold room (16 °C) in sealed plastic vessels covered with aluminum foil to prevent light degradation.

### Characterization

#### High-Performance Liquid Chromatography Mass Spectrometry (HPLC-MS)

To trace the different flavonoid components in ROD, HPLC analysis was performed using an Agilent HPLC-VWD (Santa Clara, CA) equipped with a diode array UV detector. Detection wavelengths were 254, 420, and 530 nm. Components of the mixture were separated with a Phenomenex Luna C18 column (3 μm, 100 Å, 150 mm × 4.6 mm) using a 0.8 mL/min flowrate with mobile phases A (15% MeOH in acetonitrile) and B (3% formic acid in MQ-water). The concentration of the sample was 2 mg/mL, and the injection volume was 10 μL. The gradient elution started at 97% B and was decreased to 88.5% B within 40 min, kept at 88.5% B for 10 min, and decreased to 84.5% B within 20 min, whereafter B was decreased from 84.5 to 77.0% in 15 min. At the end of the run, B was returned to 97% within 5 min where the column was equilibrated for 3 min before each run. A commercial standard was used to identify quercetin by comparison of its retention time and UV spectrum.

Also, LC-HRMS analysis was performed for the identification of the mixture components with an Agilent 1260 HPLC-QTOF-MS (D402) (Santa Clara, CA) equipped with an electrospray ionization (ESI) interface in positive ion detection mode. The samples were separated with the Phenomenex Luna C18 column (3 μm, 100 Å, 150 mm × 4.6 mm). The flowrate was 0.5 mL/min with mobile phases A (3% formic acid in MQ-water) and B (15% MeOH in acetonitrile). The concentration of the sample was 1 mg/mL, and the injection volume was 10–15 μL. In phase A, the gradient elution started at 97% and was decreased to 88.5% within 50 min, then kept at 88.5% for 15 min, and decreased to 86.5% within 15 min, and further 84.5% in 10 min, whereafter A was decreased from 84.5 to 77.0% in 20 min, and at the end was kept at 77.0% A for 10 min. The ESI source settings were the following: capillary voltage 3500 V, source temperature 300 °C, drying gas 12 L/min (nitrogen), nebulizer pressure 25 psi, fragmentor 150 V, skimmer 65 V, and octopole RF 500 V. Data acquisition (2 Hz) in the profile mode was obtained using MassHunter workstation software version 7.0 (Agilent Technologies). The data were collected at a mass range (*m/z*) of 100–1500. Reference mass correction was performed with a continuous infusion of purine (*m/z* 121.0509) and hexakis(1H,1H,3H-tetrafluoropropoxy)phosphazine (*m/z* 922.0098) (Agilent Technologies) for accurate mass calibration and for ensuring reproducibility. The mass accuracy of the instrument using external calibration was specified to be ≤3 ppm.

#### Nuclear Magnetic Resonance (NMR)

NMR spectra of both ROD and purified pectin were acquired with a 400 MHz (Avance III 400) Bruker instrument equipped with a BBFO probe. The DMSO (δC, 39.5 ppm; δH, 2.49 ppm) and 1.3.5-trioxane (δC, 93.7 ppm; δH, 5.12 ppm) were adopted as internal references for chemical shifts of ROD and pectin, respectively.^28^^1^H NMR spectra of ROD, dissolved in the DMSO-*d*_6_/pyridine-*d*_5_ (4:1 v/v) solvent, were acquired using a relaxation delay (d1) of 1 s, spectral width (SW) of 16 ppm, 1400 transients, and 64k data points. ^13^C NMR spectra was acquired with a 30° pulse using d1 2s, SW 236 ppm, 9950 transients, and 64k data points. Phase-sensitive 2D ^1^H–^13^C HSQC spectra were acquired with SW 13.0 ppm for ^1^H and 165.0 ppm for ^13^C using d1 2s, 100 transients, 256 t1 increments, d24 delay of 0.89 ms, and 1k data points. An adiabatic version of the HSQC experiment was used (hsqcetgpsisp.2 pulse sequence from the Bruker Library). Topspin 4.0.3 (Bruker) was applied for spectra processing. Chemical shifts of quercetin and pectin were assigned based on the quercetin spectra (95% HPLC, Sigma) and literature values.^29^

#### Fourier Transform Infrared Spectroscopy (FTIR)

FTIR-ATR was performed to determine the chemical composition and binding of ROD on cellulose films and textiles. The spectra were acquired using a PerkinElmer Spectrum Two spectrometer (Waltham, MA). The data were recorded over a wave number range from 500 to 4000 cm^–1^ with 10 accumulation scans and 4 cm^–1^ resolution.

#### ζ-Potential

The electrophoretic mobility of chitosan and ROD at varying pH values was measured using a Zetasizer Nano ZS90 instrument (Malvern Instruments, U.K.). The pH (2–11) was adjusted using acetic acid or NaOH. The ζ-potential data were obtained from the electrophoretic mobility data by applying the Smoluchowski model. All of the measurements were performed in triplicate, and the results were reported as an average followed by respective standard deviations.

#### In Situ Real-Time Adsorption Kinetics of ROD and Mordants Studied by Quartz Crystal Microbalance

The adsorption kinetics of ROD onto CNF thin films using either chitosan or a ferrous metal salt as a mordant was monitored at room temperature using a Q-Sense E4 instrument (Q-sense, Sweden). All of the materials were injected through the chambers at a constant flowrate of 100 μL/min. To mimic the textile wet dyeing processing, the following methodology was adopted. First, a buffer solution (1% acetic acid solution adjusted to pH 4 using 1 M NaOH) was passed through the QCM-D chamber until a stable baseline was obtained. Then, either FeSO_4_ or chitosan at 0.1 g/L concentration dissolved in 1% acetic acid solution (pH 4) was injected, and the resonance frequency (5 MHz) was monitored for 30 min until the change in frequency leveled off. Finally, a ROD solution (0.1 g/L) was injected into the chambers and the change in frequency and dissipation was monitored for 90 min. Between each step, the chamber was rinsed with the buffer solution for 30 min to remove molecules or ions not strongly attached to the surface.

To represent the cotton or cellulosic fabrics in dyeing conditions, thin films of CNF were prepared on 14 mm diameter QCM-D quartz crystal resonators with gold electrodes (Q-Sense, Biolin Scientific, Sweden). The CNF thin films were prepared by spin coating of a CNF suspension.^30^ Briefly, first, QCM resonators were treated in UV–ozone plasma to remove any adsorbed organic matter. The resonators were then adsorbed with polyethyleneimine (PEI, *M*_w_ 50 000–100 000, Sigma-Aldrich) to act as an anchor polymer to CNF fibrils. PEI droplets were placed on top of the resonators, and the polymer was allowed to adsorb for 10 min before being thoroughly rinsed with deionized water and dried under nitrogen. CNF suspension, containing only the finest CNF fibrils, was then spin-coated onto resonators (4000 rpm, 1 min). Finally, the CNF surfaces were treated in a 165 °C oven for 5 min to improve the adhesion before being cleaned with nitrogen flow.

#### UV-Shielding Tests

The UV-shielding effect of ROD was evaluated using self-standing CNF films. Briefly, the CNF suspension (100 mL, 0.8 wt %) was poured on a poly(vinylidene fluoride) (PVDF) membrane filter (hydrophilic PVDF, 142 mm membrane with a 0.45 μm pore size, Dupore), and the water was removed using air pressure filtration equipment integrated with a tripod chamber (inner diameter of 12 cm, height 8.5 cm) under 2.5 bar overpressure for 45 min. The formed CNF wet film was then ambient dried at 23 °C and 50% relative humidity for 72 h under a load of 5 kg. Then, the CNF-dried film was removed from the PVDF membrane.^31^

The CNF films were dyed with ROD liquor, keeping the fiber-to-dye liquor mass ratio at 50 g/L (1:20). The dyeing was conducted with the same methodology used in the adsorption measurements. First, CNF films were immersed in 1% acetic acid solution (pH 4) for 30 min, then samples were moved to mordant solutions (chitosan and FeSO_4_ 5 g/L) for 30 min, followed by rinsing with 1% pH 4 acetic acid. Next, the samples were immersed in the dye liquor of concentrations 0.01, 0.1, 0.25, 0.5, and 1.0% of dye on the weight of the fiber (owf) for 60 min, followed by washing in 1% pH 4 acetic acid and distilled water (pH 6).

The optical transmittance between 400 and 800 nm was measured at room temperature using a diffuse reflectance accessory coupled to a UV–Vis–NIR Agilent Cary 5000 spectrometer (Agilent, CA).

#### Textile Dyeing

To remove textile auxiliaries and impurities prior to dyeing, each textile sample (10 g) was washed in 1% (v/v) acetic acid solution (pH 2.8) in a fabric-to-dye liquor mass ratio of 1:20 at 40 °C for 30 min whereafter rinsed with water.

The fabric samples were premordanted with alum, FeSO_4_, or chitosan. The premordanting and dyeing conditions are explained in Figure S1 and Table S1. Mordanting was carried out in 0.5 g/L chitosan solution in a fabric-to-liquor mass ratio of 1:20 at 80 °C for 30 min, whereas with 8 g/L alum solution and 3 g/L FeSO_4_ solution, a fabric-to-liquor mass ratio of 1:10 at 50 °C for 60 min was used. All samples were rinsed twice with water for 10 min after mordanting to remove the unfixed mordant. The dyeing with ROD liquor (0.1 g/L) was carried out in a fabric-to-liquor mass ratio of 1:20 at 50 °C for 95 min. An Original Hanau Linitest equipment (Hanau, Germany) was used to simulate the industrial winch dyeing machine.

#### Color Strength and Light Fastness of Red Onion Dyed Fabrics

Alterations of the fabric surface after premordanting and dyeing were evaluated by comparing the FTIR spectra of untreated, pretreated, and dyed fabric samples. A Bruker α-P FTIR instrument with software Bruker Opus 6.5 (Billerica, MA) was used.

The color of the dyed sample was measured as the recommendation of the color scale, CIE L*, a*, b*, and reflectance values, using a Konica Minolta (Tokyo, Japan) CM-2600d spectrophotometer (illuminant D65, CIE 10° observer). Specular Component Included (SCI) values were recorded. This type of color evaluation measures the total appearance independent of surface conditions.^32^L*, a*, and b* refer to the three axes of CIE color space: L* representing the lightness axis and obtaining values from zero for perfect black to 100 for white; a* presenting both the hue and the chroma of the red–green axis where a* > 0 describes the redness, and a* < 0, the greenness; and b* representing the blue–yellow axis where b* > 0 indicates the yellowness, and b* < 0, the blueness of the color.^32^ The total color difference, Δ*E*, between the two samples can be defined in terms of a difference in these three components (eq 1)
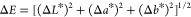
1where ΔL* = L*_1_ – L*_2_, Δa* = a*_1_ – a*_2_ and Δb* = b*_1_ – b*_2_.

The *K*/*S* value, used to determine the depth of color of dyed fabric, was calculated by the Kubelka–Munk equation (eq 2)
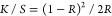
2where *K* is the absorption coefficient of the fabric to be tested, *S* is the scattering coefficient of the fabric to be tested, and *R* is the reflectance at the maximum absorption wavelengths. In this study, the reflectance values at wavelengths of 480 and 520 nm were used for calculations.

The color fastness properties of the dyed fabrics were tested according to the International Organization for Standardization (ISO). The color fastness for domestic and commercial laundering was carried out according to the ISO 105-C06:2010 standard using the AATCC detergent (without optical brightener, pH 10) or, for comparison, the AATCC detergent pH was adjusted to neutral, pH 7, to study the effect of the detergent’s pH on the fabric color in washing. The A1S washing method at 40 °C for 30 min was followed, with the DW multifiber test fabric and 10 steel balls. An Original Hanau Linitest machine (Hanau, Germany) was used to run the laundering tests.

The light fastness of the dyed fabrics was tested following the ISO 105-B02:2014 standard, Method 2, using the James Heal TruFade 200 equipment (James Heal, Halifax, U.K.) with a xenon arc lamp. To evaluate the light fastness, an eight-step blue scale was exposed to the samples. The rubbing fastness (dry) was tested according to the ISO 105-X12:2016 standard.

## Results and Discussion

### Chemical Characterization of the Red Onion Dye and Dyed Fabrics

**Figure 2:**
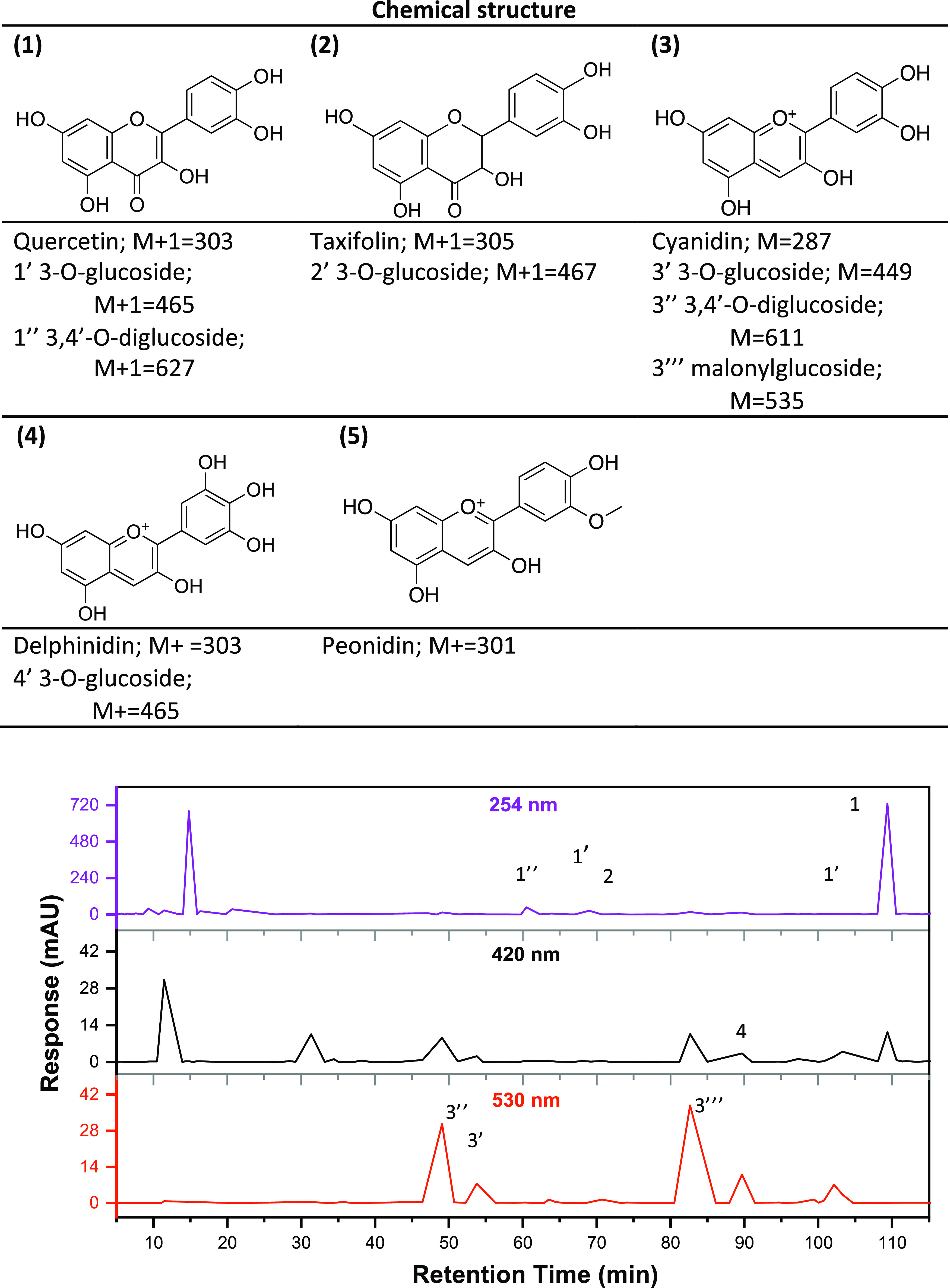
HPLC chromatograms and qualitative identification of the main flavonoids in ROD detected at 254, 420, and 530 nm.

The concept of directly employing natural plant extracts after extraction in hot water is a cost-effective way to acquire natural dyes. However, these natural dye extracts are not pure substances; instead, they contain a complex mixture of flavonoids and other water-soluble compounds. To identify the main components of the ROD mixture, HPLC and high-resolution ESI-QTOF-MS as well as NMR were employed. Figure 2. comprises the chemical structures of the main flavonoids, as well as the HPLC chromatograms of ROD at wavelengths 254, 420, and 530 nm.

The chromatogram obtained at 254 nm shows two major absorption peaks, of which the latter one, at retention time (RT) 108.3 min, was also detected at 420 nm. This signal is attributed to flavonol quercetin (**1**) as also confirmed by the correlation to the RT of the pure quercetin standard (data not shown). The NMR spectra (Figure S2a–c) also confirmed quercetin as the main phenolic compound in the ROD. Carbohydrate analysis of onion extracts revealed glucose and pectin galacturonic acid as the main carbohydrates (Figure S3 and Tables S2 and S3). In ESI-QTOF-HRMS positive ion mode, the intense peak at RT 108.3 min with [M + H]^+^ 303.0508 was found, and in comparison to the quercetin [C_15_H_10_O_7_] + H^+^ calculated exact mass 303.0499, the mass error was only 3 ppm, further confirming the presence of quercetin. The HRMS analysis revealed the mono- and diglucosides of quercetin at 59.7, 67.4, and 103.8 min. Another flavonoid compound with the observed mass [M+H]^+^ 305.0656 could be detected at 71.1 min in small intensity and was interpreted to be taxifolin (**2**) [C_15_H_12_O_7_] + H^+^ with a calculated mass 305.0671 and mass difference of 1.6 ppm. The chromatogram obtained at 530 nm revealed many small peaks suggesting the presence of several compounds that absorb in the long wavelengths, mainly anthocyanins such as cyanidin (**3**).^33^ We note that some of the characteristic peaks of anthocyanins also absorb at 530 and 420 nm in the UV region.

Red onion (*A. cepa*) skin has previously been reported to be composed of flavonoids, mainly flavonols, and anthocyanins.^34−40^ Despite the complex formulation of ROD, anthocyanins and quercetin should play a key role in color formation. Anthocyanin is a chromophore structure that can create a red, blue, or purple color depending on the pH and temperature conditions. Quercetin (**1**) and its derivatives (mono-, di- and triglucosides, e.g., quercetin 3-O-glucoside and quercetin 7,4′-O-diglucoside) are the most abundantly occurring compounds in plant extracts and are usually attributed to the formation of yellow color.^41,42^ Fossen and co-workers (1996) studied the red onion cultivar, Red Baron, the same as that used in this study, and reported cyanidin (**3**) derivatives, such as cyanidin 3-glucoside, cyanidin 3-(3″-malonylglucoside), cyanidin 3-(6″-malonylglucoside), and cyanidin 3-(3″,6″-dimalonylglucoside), as the major anthocyanins in the extract.^33^ At 530 nm, two strong peaks at retention times 47.8 and 82.5 min were observed. According to HRMS, we suggest that these peaks originate from cyanidin 3-O-glucoside [M]^+^ 449.1084 and cyanidin 3-O-malonylglucoside [M]^+^ 535.1103. On the basis of the UV spectra at retention times 86.3 and 88.0 min with observed [M]^+^ ions 303.0505 and 465.1037, we suggest that these originate from other anthocyanin-type compounds as delphinidin (**4**) derivatives, in accordance with recent reports.^43^ Peonidin-glucoside (**5**) derivatives were not detected in our ROD, even though they have been found in other cultivars.^44^ However, due to the lack of standards, these compounds could not be more reliably identified or quantified.

The analyses showed that ROD is a mixture of several components, both uncharged neutral flavonols and positively charged anthocyanins at acidic pH, together with their glucoside derivatives. In natural dye extracts, formation of supramolecular complexes between anthocyanin pigments and flavonoid copigments, the so-called copigmentation, has been shown to have an important color-stabilizing and modulating effect.^45^ In addition, the ROD also contained high amounts of pectin with galacturonic acid functionalities. These all can affect the ROD’s adsorption behavior and activity during dyeing.

The FTIR spectrum of freeze-dried ROD powder and dyed fabrics is shown in Figure S4. In the spectrum of ROD (Figure S4a), the broad absorption band observed at 3246 cm^–1^ is due to the hydroxyl group stretching vibrations, while the band at 2924 cm^–1^ relates to CH_2_–CH (C–H stretch), and a nonconjugated carbonyl group (C=O stretch), which is indicated by a shoulder at 1725 cm^–1^. In the fingerprint region, the bands at 1600 and 1510–1450 cm^–1^ indicate the presence of conjugated, aromatic carbon–carbon double bonds and the aromatic ring stretching (C=C–C), while the C–C bond stretching and phenolic C–O stretching are observed as several bands at 1200 cm^–1^, typical for flavonoids. Several absorption bands at 1500–1350 cm^–1^ originate from varying C–H bending vibrations. The band at 1015 cm^–1^ suggests glycosidic C–O stretching vibrations, and the peaks in the area from 770 to 630 cm^–1^ are attributed to ring torsion and =C–H out-of-plane bendings. Together, these bands suggest the presence of structures typical for the different flavonoid backbones.

### Effect of Chitosan on In Situ Adsorption of ROD on CNF as Studied by QCM-D

**Figure 3:**
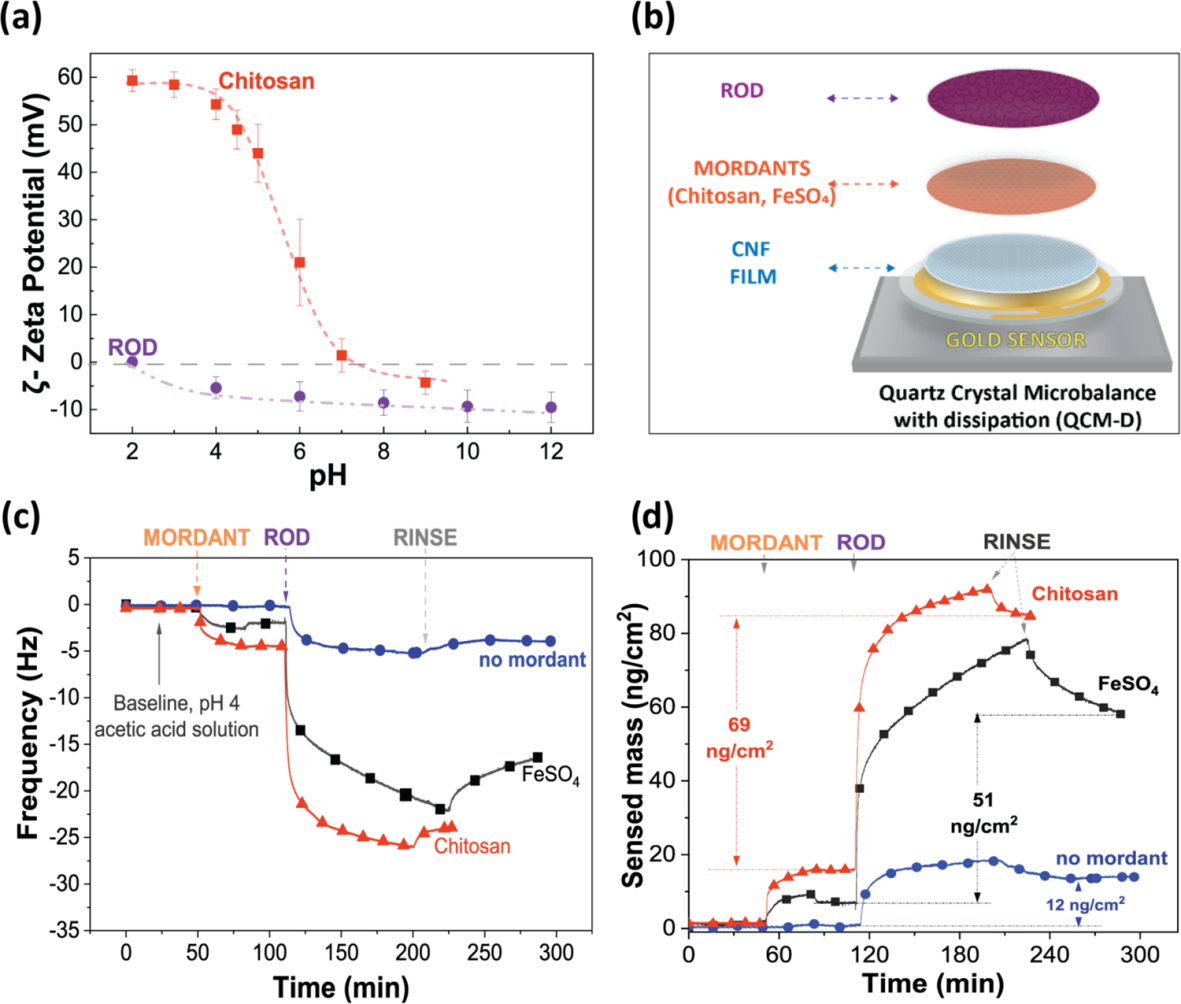
(a) ζ-Potential of chitosan and onion extract solution as a function of pH (zero potential is marked as a gray line). (b) Model of ROD adsorption onto the CNF thin films using FeSO_4_ or chitosan as mordants. (c) Change in oscillation frequency (fifth overtone) as a function of time of ROD adsorbed onto CNF with FeSO_4_ and chitosan as mordants. Mordants, the buffer for rinsing, dye, and second rinsing were applied at *t* ≅ 60, 90, 120, and 210 min, respectively. (d) Sensed mass as a function of time and the remaining ROD after rinsing calculated by Sauerbrey’s equation.

While there is a growing interest toward biocolorants and natural mordants, very little is known about their adsorption mechanisms. To address this knowledge gap, we employed in situ adsorption measurements using QCM-D. Charge density and distribution may play a critical role in the binding between natural dyes and cellulosic fibers when using polyelectrolytes as mordants. Hence, the charge densities of chitosan and ROD were qualitatively estimated by measuring the ζ-potential at different pH values (Figure 3a).

The chitosan solution exhibited positive ζ-potential values in the acidic environment, but when the pH became neutral, a zero-net charge was observed causing the material to precipitate. This is the expected behavior for chitosan since its primary amine groups are gradually deprotonated as the pH increases. ROD was neutral at very acidic conditions (pH 2) and carried a low negative charge at pH 4 and above. To preserve ROD from very acidic conditions while maintaining the strongest interaction between ROD and chitosan (based on the highest ζ-potential difference), pH 4 was chosen as the optimal setting for adsorption measurements in QCM-D. The CNFs prepared from the same pulp and using a similar procedure as used here have been reported to carry a low negative charge.^46,47^ Furthermore, preliminary results showed that ROD was slightly better absorbed to CNF model films at pH 4 (Figure S5), and previous studies also supported a mildly acidic environment as an optimal condition for dyeing using plant-extracted natural dyes.^48−51^

ROD adsorption onto CNF thin films (Figure 3c) was performed stepwise. First, the CNF thin films were equilibrated in the buffer until a stable baseline was achieved, then each mordant (chitosan or FeSO_4_) was injected into the chambers, allowed to adsorb until reaching a plateau, after which excess of mordant molecules was removed via rinsing with the buffer. Then, the ROD was finally allowed to be adsorbed, and in the last rinsing step, unbound ROD was removed. In this way, adsorption of mordants and dyes could be separately monitored and the textile dyeing method simulated in detail (Figure 3b).

The QCM-D analysis revealed that the direct adsorption of ROD onto CNF was low compared to the mordanted samples. Figure 3c depicts the adsorption curves of ROD onto CNF thin films as a function of time. The direct adsorption of ROD onto the CNF film, without mordanting, showed a decrease in the oscillation frequency as soon as the ROD solution was injected into the chamber indicating that some dye molecules were adsorbed to the CNF surface reaching a plateau within 30 min. The average shift of the frequency was −5.3± 0.7 Hz, suggesting that the adsorption was very low. In contrast, a significantly higher ROD adsorption was observed when using mordants. The FeSO_4_ and chitosan mordants had similar adsorption behavior. First, as soon as the mordants were injected, the oscillation frequency shifted to −2.3± 0.5 and −4.5± 1 Hz, respectively, reaching a stable signal within 30 min meaning that both mordants rapidly adsorbed onto the CNF films (Figure 3c) but the adsorbed mass was low. Then, a first rinsing was employed (*t* ≅ 90 min), and only FeSO_4_ showed a slight increase in the frequency (0.5 ± 0.1 Hz) caused by the removal of ions not bound strongly enough to the CNF surface. No increase in frequency was observed upon rinsing after chitosan adsorption. As soon as ROD was injected into the CNF coated with mordants, a rapid decrease in frequency was detected within 10 min, revealing rapid and significant adsorption of ROD to the mordant-coated cellulose. The adsorption rate of ROD gradually decreased and did not reach a plateau in the evaluated time range, and the maximum frequency changes (Δ*f*) registered for FeSO_4_ and chitosan-mordanted samples were −21 ± 3 and −26 ±2 Hz, respectively. It should be noted that the adsorption rate to FeSO_4_-coated CNF was slower than that to chitosan-coated CNF suggesting different adsorption mechanisms. Finally, the ROD compounds that were not strongly bound to the mordant-coated CNF substrate were removed during the second rinsing step (*t* ≅ 210 min) as detected by the increase in Δ*f*, from −21 ± 3 to −16 ± 7 Hz for FeSO_4_ and from −26 ± 2 to −24 ± 3 Hz for chitosan. For a convenient comparison of the systems, sensed mass curves (Figure 3d) were obtained employing the Sauerbrey equation.^52^ The sensed adsorbed mass values of ROD to pure CNF, FeSO_4_, and chitosan-mordanted CNF samples after rinsing with acetic acid were estimated to be 12, 51, and 69 ng/cm^2^, respectively. Interestingly, the adsorption of chitosan led to a higher adsorbed amount of ROD compounds compared to FeSO_4_. Chitosan not only improved the dye uptake but also firmly bound ROD to CNF films, demonstrating its efficiency as a mordant for phenol-based compounds, such as ROD.

**Figure 4:**
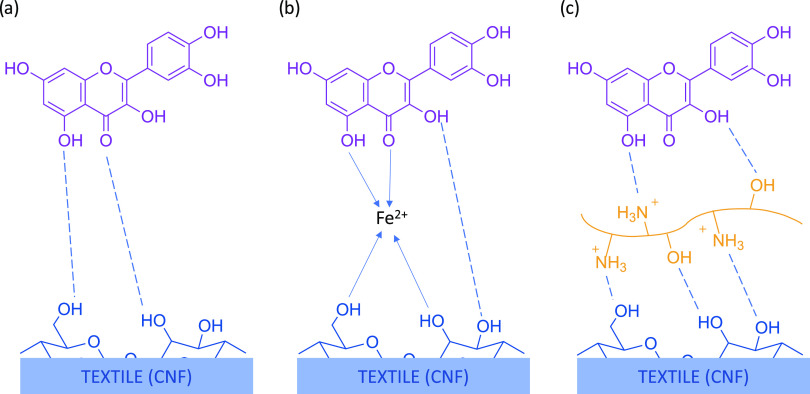
Schematic (not to scale) of possible interactions between CNF (QCM-D model film), mordants (FeSO_4_, chitosan), and a flavonoid compound as an example in ROD at pH 4; (a) direct absorption, (b) metal mordanting, (c) chitosan as the biomordant. The dashed lines indicate hydrogen bonding, and the arrow lines, coordinate bonding.

There is a weak electrostatic repulsion between cellulose and the ROD, and the only driving force for adsorption is probably a slightly poorer affinity between the phenolic compounds and water than between them and cellulose. Hence, slight adsorption is observed even in the absence of mordants, possibly arising from the uptake of the positively charged anthocyanins at pH 4 in ROD as minority components. After adsorption, van der Waals interactions may hinder desorption. In mildly acidic solutions, the anthocyanins exist in multistate equilibrium between the red flavylium cation, its hydrated colorless hemiketals, and the purple neutral quinone methide tautomers that are formed by deprotonation of the most acidic OH group.^53^ Furthermore, the noncovalent copigmentation interactions of the anthocyanin planar polarizable surface with flavonoids, e.g., quercetin and its derivatives, have been found to be important for the stabilization of the colored species by blocking the competitive hydration of the flavylium cation.^54^ Hence, the neutral copigmentation complexes are assumed as the main pigments in ROD. The cationic chitosan introduces electrostatic attraction between the electronegative ROD and cationic chitosan. The driving forces for adsorption are most probably the release of counterions and release of water molecules from around the slightly hydrophobic phenolic compounds.^55,56^ The difference in the adsorption kinetics of ROD onto FeSO_4_ and chitosan-coated samples suggest a different adsorption mechanism, as shown in Figure 4. We suggest that the complexation with metal ions is a slower process than the adsorption driven by the increase in entropy due to the release of counterions in the case of adsorption onto a polyelectrolyte. Once adsorbed, both the strong electrostatic interactions with chitosan, van der Waals forces, and the metal complex restricts the desorption of ROD from the mordanted substrates. The observed rapid adsorption of both mordants and dyes suggests that dyeing times could be shortened.

In sum, natural dyes derived from plants contain phenolic groups that can exhibit anionic characteristics depending on the pH environment. In a low acidic environment, the CNF also has a negative surface potential due to the presence of carboxylic acid groups from the oxidation of the primary hydroxylic sites during the pulping and bleaching processes.^57^ When immersed in aqueous solutions, the cellulose surface becomes slightly negatively charged creating an unfavorable environment for natural dye compound adsorption as demonstrated by very low adsorption of ROD on unmordanted CNF. When in acidic pH (below 4), carboxyl and hydroxyl groups on cellulosic fiber surfaces are mostly protonated, decreasing the electrostatic repulsion and enabling the dye molecules to come close enough for attractive van der Waals forces and hydrogen bonds between the fiber and dye molecules and creating a more likely condition for dye molecule adsorption onto CNF fibers (Figure 4a).

The presence of electron-rich carbonyl and hydroxyl groups in phenolic compounds in ROD allows the formation of coordination complexes with metal ions as mordants anchored to the CNF substrate (Figure 4b).^14^ A strong binding may also occur when chitosan is used as the mordant (Figure 4c). Chitosan is a renewable polymer with positively charged groups when in acid conditions, where also ionic interactions may take place binding the carboxylic acid groups in CNF to chitosan, supported also by hydrogen bonding.

### FTIR Characterization of Dyed Fabrics, Their Color, and Color Fastness Properties

Cotton fabrics were dyed with ROD using conventional metal ions (FeSO_4_ and KAl(SO_4_)_2_), as well as chitosan as mordants, to correlate the in situ adsorption measurements using model systems to real scale dyeing.

FTIR spectra of untreated, as well as chitosan mordanted and dyed cotton fabrics, are shown in Figure S4b. Signals from the cotton fabric dominate all of the spectra and show typical FTIR profiles of cellulose.^48^ From the chitosan-treated fabric (red line, Figure S4b, highlighted range), the amino group C–N stretch can be seen as a small peak at 1020 cm^–1^.

The strong peaks at around 1015 cm^–1^, particularly at 1057, 1031, and 986 cm^–1^ (Figure S4b), indicate C–O stretching, originating from the underlying cellulosic fabric, and appear much stronger compared to the FTIR spectrum of ROD (Figure S4a). The absorption bands at 600 cm^–1^, originating from the out-of-plane C–H bending, are strong, and they can be attributed to carbon–hydrogen bonding in the cellulose structure. FTIR spectra reveal that chitosan as a premordanting treatment stabilizes the chemical structure of the fabric–mordant–dye system (black line, Figure S4b), which is indicated by the lower intensity of the spectrum compared to untreated fabric (red and blue lines, respectively, Figure S4b).

**Figure 5:**
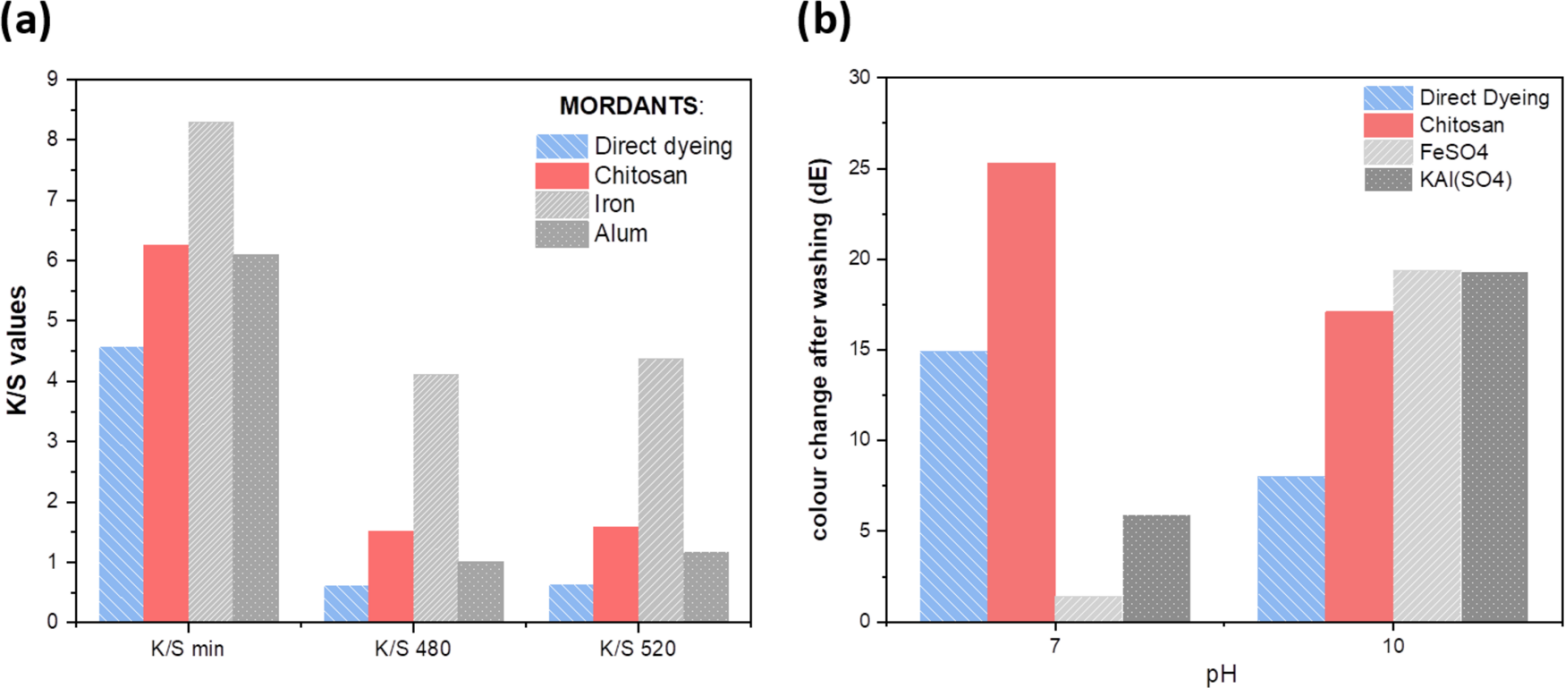
(a) Color depth expressed as *K*/*S* at minimum reflectance values, reflectance at 480 and 520 nm of the ROD-dyed cotton fabrics demonstrating the efficiency of chitosan and FeSO_4_ as mordants; the higher the value, the stronger the color. (b) Color change after washing (Δ*E*) between the untreated and washed fabric in two pH conditions; the lower the value, the least change of color in washing.

In textile applications, color depth is typically expressed as a ratio of absorption and scattering coefficients, also known as *K*/*S* values. Figure 5a depicts the *K*/*S* values for dyed cotton fabrics dyed with ROD and mordants chitosan, iron(II), and aluminum(III) at pH 4 optimum conditions and the color change values (Δ*E*) after washing tests at pH 7 and 10.

**Figure 6:**
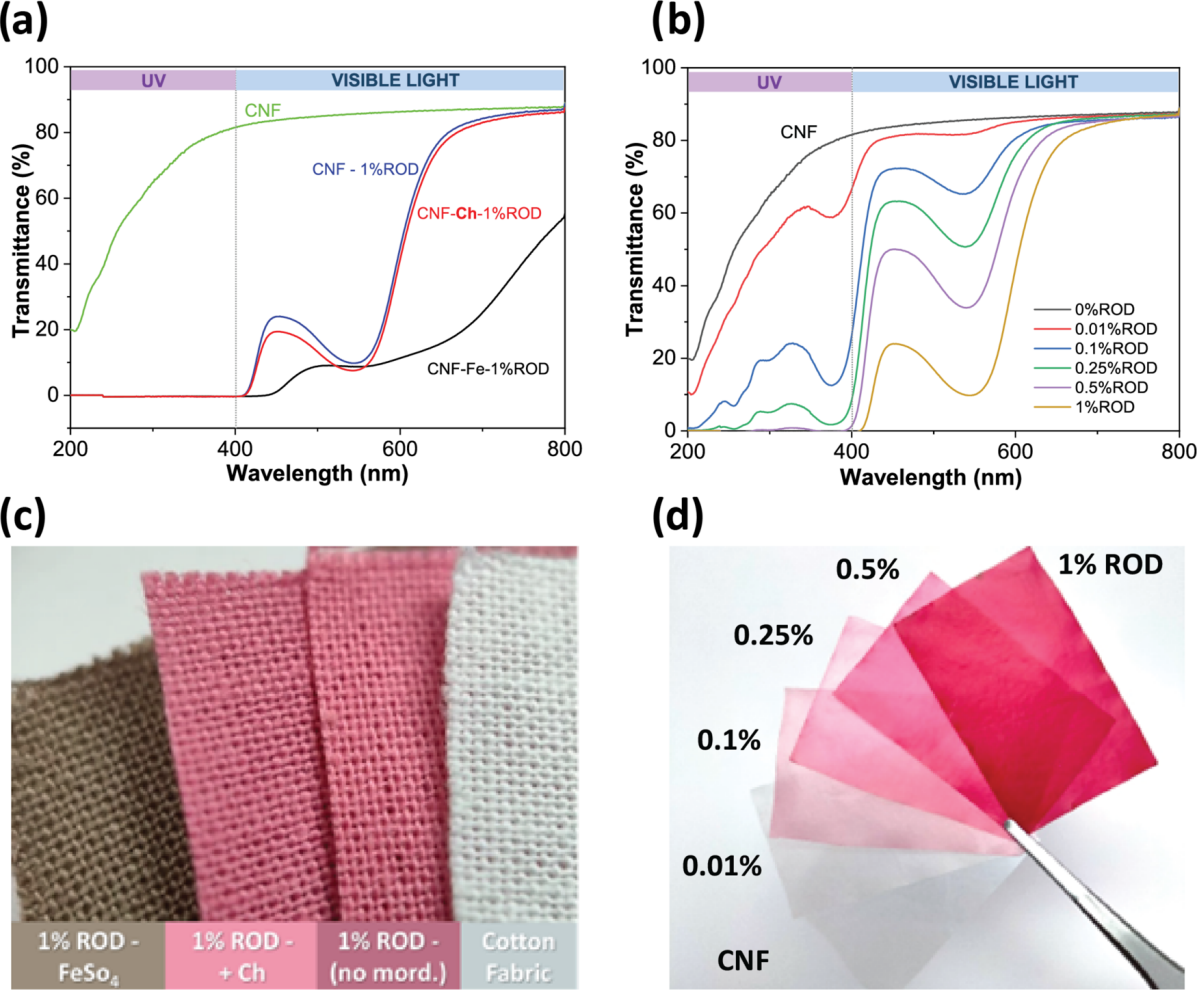
UV–vis light transmittance spectra of (a) the CNF film, CNF-dyed samples, no mordanting (1% ROD), FeSO_4_ (1% ROD–Fe), and chitosan (1% ROD–Ch) as mordants. (b) CNF-dyed films with a variety of ROD concentrations (from 0.01 to 1% owf); 0.5% owf is the minimum amount to completely block UV. (c) Photograph of 1% ROD-dyed cotton fabrics, chitosan retains the original ROD color sightly deepening it. (d) 0.01–1% owf dyed film CNF films for UV-shielding tests.

Figure 5a reveals that chitosan mordanting increases the dye absorption considerably compared to direct dyeing and even compared to the use of alum as the mordant, corroborating with previous studies^58^ and the QCM-D results in Figure 3. The color formed with alum was green as shown from the a* value less than 0 (Table S4). In contrast to the QCM-D results, the dyeing experiments indicate that the FeSO_4_ mordant brings about the strongest color of ROD as revealed by the highest *K*/*S* values. This is apparent as iron(II) forms a metal–organic chromophore complex (coordination bonds, Figure 4b), which brings about the increase in absorption coefficiency (compared to chitosan which is a polymer), thus increasing the color strength. However, it is worth mentioning that the obtained color of the Fe(II)-ROD complex is green (Table S4, L* 37.46, low, indicating dark color; negative a* indicating green), while chitosan retains the original pink shade of ROD (Table S4, L* 60.29; positive a* indicating redness, and Figure 6c). Further, the color depth value (*K*/*S*) is based on optical methods while QCM-D reflects adsorbed mass, which may also contain colorless compounds, and this explains part of the difference. Also, aluminum(III) forms a coordination complex with ROD and brings about the green color (Table S4) as indicated by the negative a* value. Hence, we note that the electrostatic interactions, van der Waals and hydrogen bonding do not change the color of the dye while coordination complexation does.

The color difference after washing (Δ*E*) (Figure 5b) reveals that the ability of chitosan to stabilize the color when subjected to alkaline laundering pHs is lower than for the metal ions, especially around neutral conditions. The greatest Δ*E* values were observed after washing at pH 10 due to the phenolic OH-groups of the natural dye that ionize, causing a bathochromic shift to the absorption spectrum and a visible change of the observed color from red to green.

When considering the staining results, it was observed that chitosan received higher values for staining than the nonmordanted samples. The color fastness values for washing and rubbing were similar to metal mordanted samples revealing that chitosan increases the adsorption of the dye onto the fiber and, thus, produces compatible results with metal mordants. However, the challenge remains in stabilizing the color chromophore, which is sensitive to light and pH changes (Figure S5c). The iron ion stabilizes the chromophore and color resulting in slightly higher values in color fastness tests and especially increases light fastness stability (Table S4), generally taken at moderate levels. The electrons of the iron ion become part of the chromophore, and the observed color changes to green. The EU eco-labeling for the light fastness of textile dyes determines the value of 4, which can be reached with iron mordanting (Table S4).^5^

### UV Shielding

Aside from color, another exciting feature of natural colorants like ROD is the functionalization of fibers or other cellulosic materials to produce added-value protective textiles or films. To evaluate the UV-shielding potential of ROD on cellulosic materials, CNF self-standing films were dyed with ROD with and without mordanting (Figure 6d). Figure 6a comprises the UV–vis transmittance spectra of neat CNF films and CNF films dyed in 1 wt % ROD samples (CNF-1% ROD) using FeSO_4_ (CNF–Fe-1% ROD) and chitosan (CNF–Ch-1% ROD) as mordants.

The remarkable UV-blocking capability of ROD compounds became evident in all samples dyed with 1% ROD since the 400–200 nm wavelength range, comprising both UV-A and UV-B radiation, was completely suppressed. This outcome is attributed to the structural diversity of phenolic compounds in the ROD that possess UV-absorbing functional groups.

Although the mordants FeSO_4_ and chitosan did not cause any noticeable changes in the UV region since the transmittance was zero in the UV region for ROD-dyed samples with and without mordants, a few authors have previously reported an increase in the UV-A and UV-B shielding effect in different textiles using natural dyes from different plant sources when they have been treated with chitosan.^18,59^ The second experiment with direct dyeing of the CNF films with ROD in a variety of concentrations (Figure 6b) revealed that the concentration of 0.5% owf of ROD was already enough to obtain almost complete UV blocking, suggesting that lower ROD concentrations are enough and the effect of mordants on the UV-shielding efficiency may be clearer when using very low ROD concentrations. The advantage of the low dye concentration is that the film remains translucent, which can be beneficial in packaging applications.

Color hues and transparency changes in the dyed films can also be inferred in the UV–vis transmittance spectra. Chitosan-mordanted samples had nearly the same spectra and maximum transmittance as CNF-1% ROD and neat CNF films (86%). The absorption band detected at 530 nm is attributed to the anthocyanin chromophores with flavylium ions at low pH conditions, which give the samples their purplish color. On the other hand, the complex formed between the metallic ions and ROD compounds in CNF–Fe-ROD samples completely changed the color and inevitably increased the light scattering, reducing the maximum transmittance from 85 to 45% (Figure 6c).

The color stability of the dyed fabrics for UV and visible light, washing, and rubbing were tested according to the ISO 105-B02, C06, and X12 standards, respectively, the results of which are reported in Table S4. The light fastness, washing fastness, and rubbing fastness of the dyed fabrics receive values from moderate to good. The dye extraction and dyeing processes were repeated numerous times on a laboratory scale and the results have been repeatable.

## Conclusions

By modeling the traditional dyeing process in QCM-D, this study for the first time analytically evaluates the interaction mechanisms underlying dye absorption and binding to cellulosic substrates, expanding the fundamental understanding of natural dye adsorption. The adsorption of ROD onto CNF thin films and the ζ-potential measurements revealed pH 4 as optimal for ROD adsorption to cellulose. Adsorption curves and dyeing experiments demonstrated that chitosan, due to its cationic nature in acid pH, can significantly increase dye adsorption onto cellulosic films or fabrics, cementing its potential as a natural mordant. The adsorption of ROD onto chitosan-mordanted CNF films was very rapid and metal salts could be avoided. Efficient UV blocking required furthermore only 0.5 and 1% owf of the concentration of the dye and mordant solutions, respectively. Importantly, chitosan retained the original dye color, with a slight shift to red/pink shades, whereas FeSO_4_ and alum-mordanted samples completely changed the obtained color to green shades due to metal ion–dye complexation. The combination of plant-based natural dyes and mordants derived from biomass side streams, such as chitosan, is a promising approach to fulfill the textile industry in seeking for alternatives to hazardous chemicals and auxiliaries in their processes. Studies show that agricultural and food processing wastes provide adequate streams for valorization of natural dyes and biomordants, especially when applied on a niche scale.^4^

## Supporting Information Available

General procedure (Figure S1); experimental setup (Table S1); 1 and 2D NMR spectra of ROD (Figure S2); NMR spectra of pectin (Figure S3); mass balance of HWE (Table S2); chemical characterization of pectin (Table S3); FTIR spectra of ROD and dyed fabrics (Figure S4); change in oscillation frequencies in ROD in different pH, maximum sensed masses, and visual appearances of ROD in varying pH and on standing (Figure S5); and CIE *L**, *a**, *b** color coordinates and visual color, *K*/*S* values, washing fastness, and rubbing fastness of the dyed cotton fabrics (Table S4) (PDF)

## Abbreviations

ROD: red organic dye
FeSO_4_: ferrous sulfate
KAl(SO_4_)_2_: alum, potassium aluminum sulfate
CNF: cellulose nanofiber
DCM-D: quartz crystal microbalance with dissipation
HPLC-MS: high-performance liquid chromatography combined with mass spectrometry
FTIR: Fourier transform infrared spectroscopy
UV–Vis: ultraviolet–visible spectrophotometry
ISO: International Organization for Standardization
