# Supplementary material for: In Situ Adsorption of Red Onion (Allium cepa) Natural Dye on Cellulose Model Films and Fabrics Exploiting Chitosan as a Natural Mordant
Source: ACS Omega. 2023 Feb 1;8(6):5451–63. doi: 10.1021/acsomega.2c06650 (PMC9933475; doi:10.1021/acsomega.2c06650)
Supplement: Supplementary file 1 — ao2c06650_si_001.pdf [file ao2c06650_si_001.pdf]

SUPPLEMENTARY INFORMATION

In situ adsorption of red onion (*Allium cepa*) natural dye on cellulose model films and fabrics exploiting chitosan as a natural mordant

Rafael Grande[a], Riikka Räisänen[b], Jinze Dou[a], Satu Rajala[b], Kiia Malinen[a], Paula A. Nousiainen[a], Monika Österberg*[a]*

[a]Aalto University, School of Chemical Engineering, Department of Bioproducts and Biosystems, Vuorimiehentie 1, 02150 Espoo, Finland

[b]University of Helsinki, Craft Science, Siltavuorenpenger 10, 00014 Helsinki, Finland

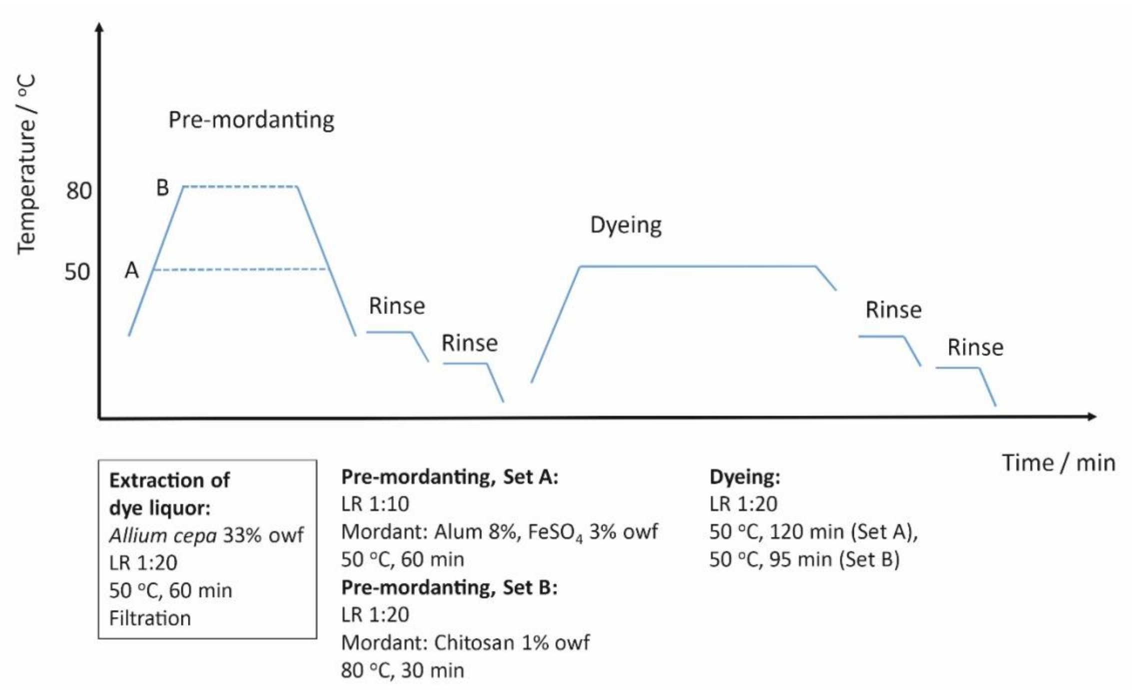

Figure S1. Procedure simulating industrial dyeing of cotton fabric with red onion (*A. cepa*) dye. owf = on the weight of the fiber.

Table S1. The pre mordanting and dyeing conditions of the experimental set-ups A and B. Mordant amount is measured by percentage on the weight of the fibre (owf).

Sample	*Pre mordanting*		*Dyeing*
	Mordant (owf)	pH	pH
A1-0	0	-	4.1
A2-Al	Alum 8%	3.5	3.3
A3-Fe	$FeSO_4$ 3%	3.6	3.9
B1-Ch	Ch 1%	3.1	3.8

a)

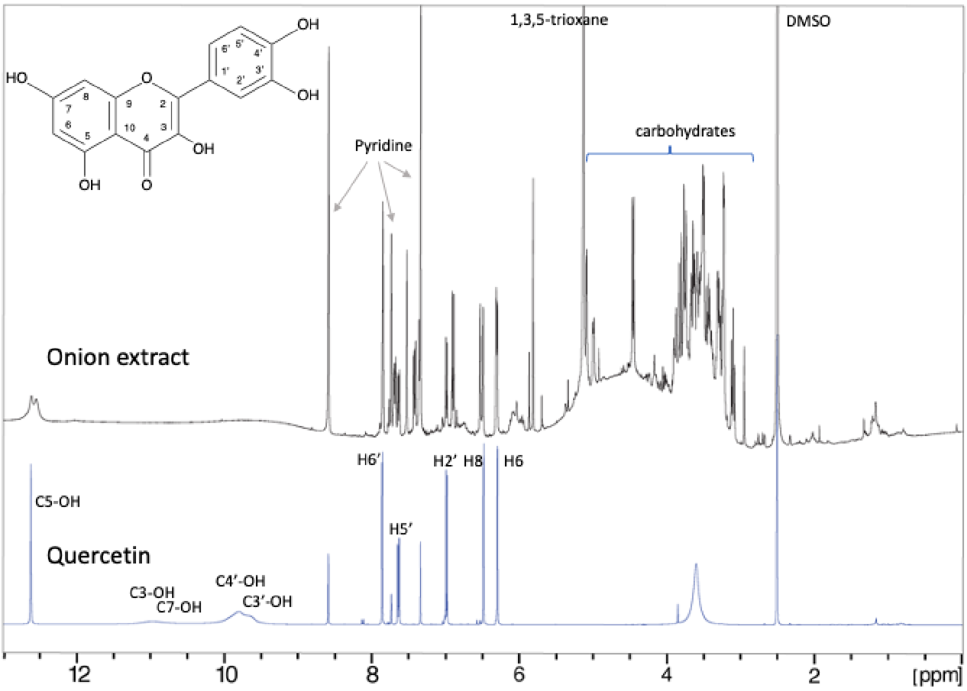
1,3,5-trioxane
DMSO
Pyridine
carbohydrates
Onion extract
Quercetin
C5-OH
H6'
H2'
H8
H6
H5'
C3-OH
C7-OH
C4'-OH
C3'-OH
12
10
8
6
4
2
[ppm]

b)

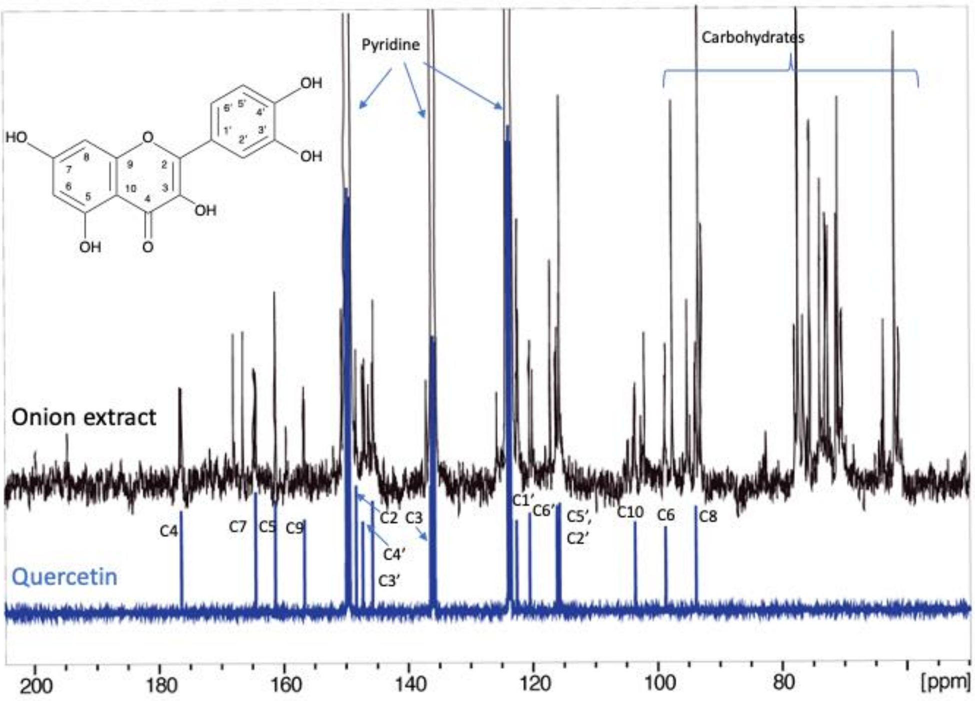
Pyridine
Carbohydrates
Onion extract
Quercetin
C4
C7
C5
C9
C2
C3
C4'
C3'
C1'
C6'
C5',
C2'
C10
C6
C8
200
180
160
140
120
100
80
[ppm]

c) d)

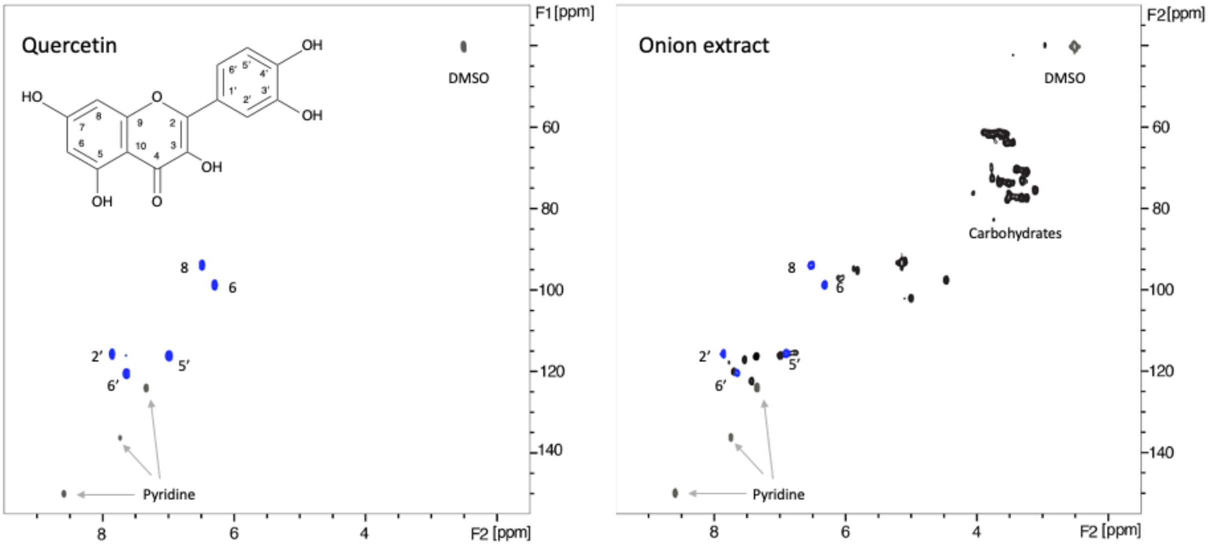

Figure S2. a) ^{1}H and b) ^{13}C NMR spectra of ROD in comparison with standard quercetin in DMSO-d6/pyridine-d5. 2D ^{1}H–^{13}C HSQC NMR spectrum (δ_C/δ_H, 30–155/1.5–9.5 ppm) with c) standard quercetin and d) onion extract in DMSO-d6/pyridine-d5.

The purified pectin (**Table S2**) was systematically chemically characterized by NMR spectroscopy and chromatography techniques (i.e., HPAEC-PAD and HPLC). The galacturonic acid (GalA) represented approximately 57% of the identified carbohydrates (**Table S3**) and this explains that the 1,4-glycosidically linked GalA unit is the dominant linkage at pectin. Specifically, in HSQC NMR spectrum of the purified pectin fraction, two strong and well resolved chemical shifts (**Fig. S3**) at δ_C/δ_H, 52.5/3.72 ppm and δ_C/δ_H, 20.9/2.11 ppm indicated the strong presence of C/H atoms of the methyl ester groups of carboxylic acid moiety of 1,4-α-D-GalpA and the acetate ester-groups of at the O2/O3-position of 1,4-α-D-GalpA. Moreover, after the two-hours saponification, the detected methanol and acetic acid are directly proportional to the released pectin esters, respectively.[1] Interestingly, these carboxylic acid–carboxylate functional groups of pectin are known to absorb dyes and metal ions.[2] The detailed structural chemical characteristic of pectin of the red onion extract is out of scope of this present study.

Table S2**.** Mass Balance of the Hot Water Extract (**HWE**) from Onion. The standard deviations are included in the parenthesis.

	mg/g extracts
Arabinose	1.0 (0.2)
Galactose	0.4 (0.1)
Glucose	121 (6.3)
Mannose	10 (1.7)
Total carbohydrates[a]	132
Quercetin[b]	53 (4.7)
Pectin[c]	203 (13)
Ash	58
Sum	447

[a] Determined by HPAEC-PAD

[b] Determined by ^{1}H NMR

[c] Gravimetric differences of the precipitates from the pectin purification step

a)

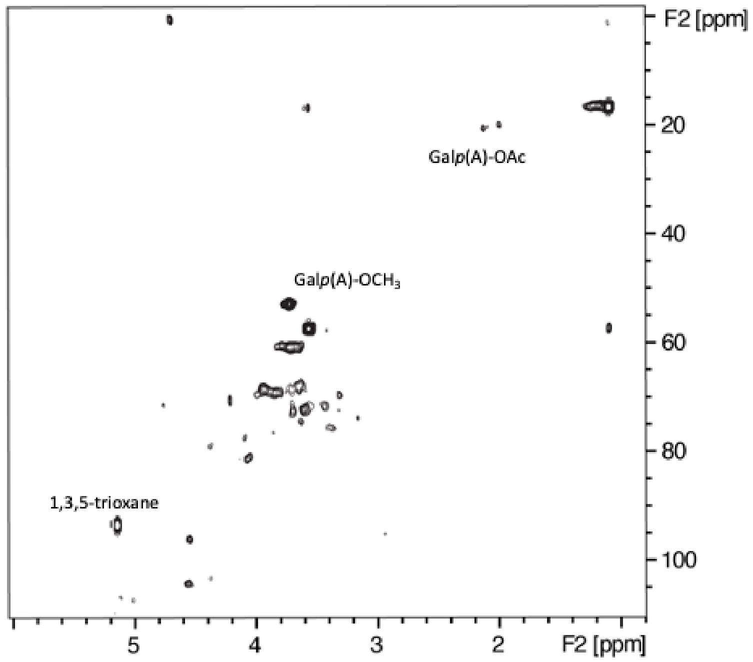

b)

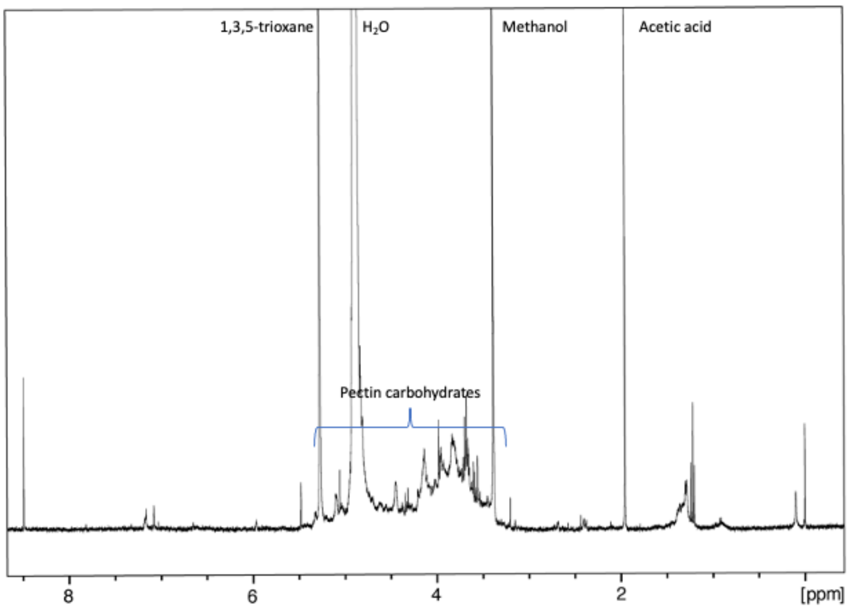

Figure S3. a) 2D HSQC NMR spectra regions (δ_C/δ_H, 0–110/0–6.0 ppm) of the purified Pectin from onion water extracts using D_2O as the solvent and b) ^1H NMR spectrum of the purified Pectin from onion water extracts treated under alkaline (NaOH 0.45 mol/l) conditions for two hours using D_2O as the solvent.

Table S3. Chemical characteristics of the purified pectin (**Table 1**) by HPAEC-PAD and HPLC. The standard deviations are included in the parenthesis.

	mg/g pectin
Arabinose	13 (1)
Rhamnose	26 (3)
Galactose	50 (5)
Glucose	127 (13)
Galacturonic acid	283
sum	499

a)

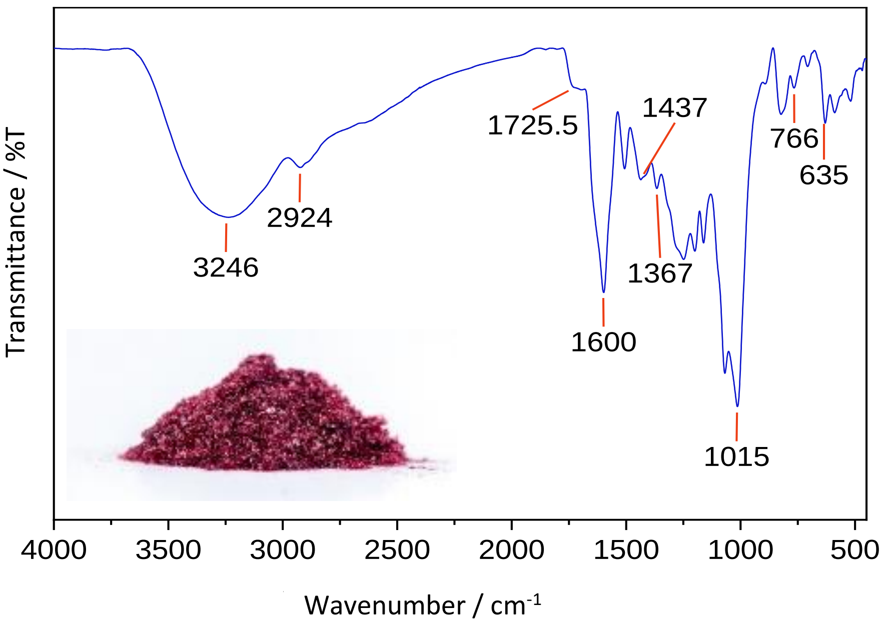

b)

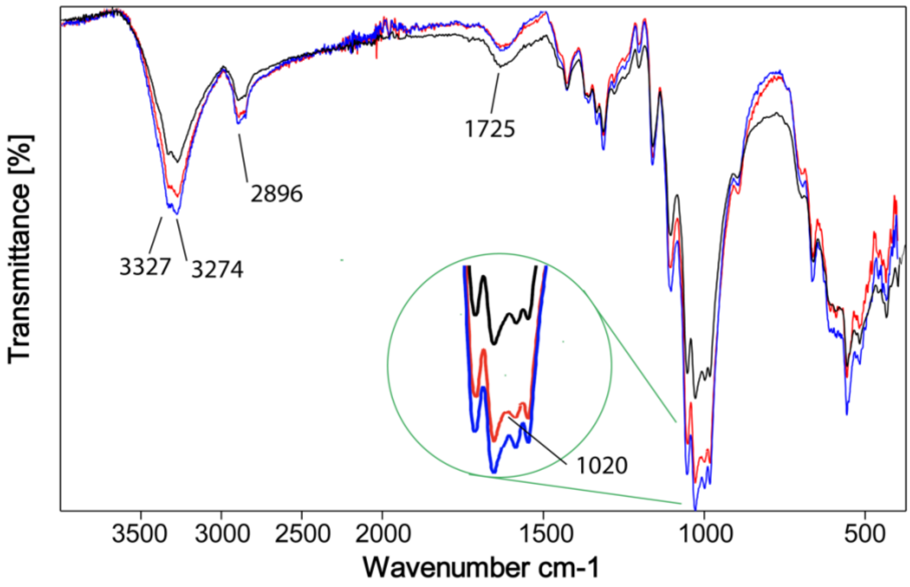

Figure S4. FTIR spectra of **a)** red onion dye (ROD), dried powder (insert) and **b)** untreated and dyed cotton fabrics. Blue line = untreated cotton fabric, red = cotton and chitosan, black = cotton with chitosan mordanting and ROD.

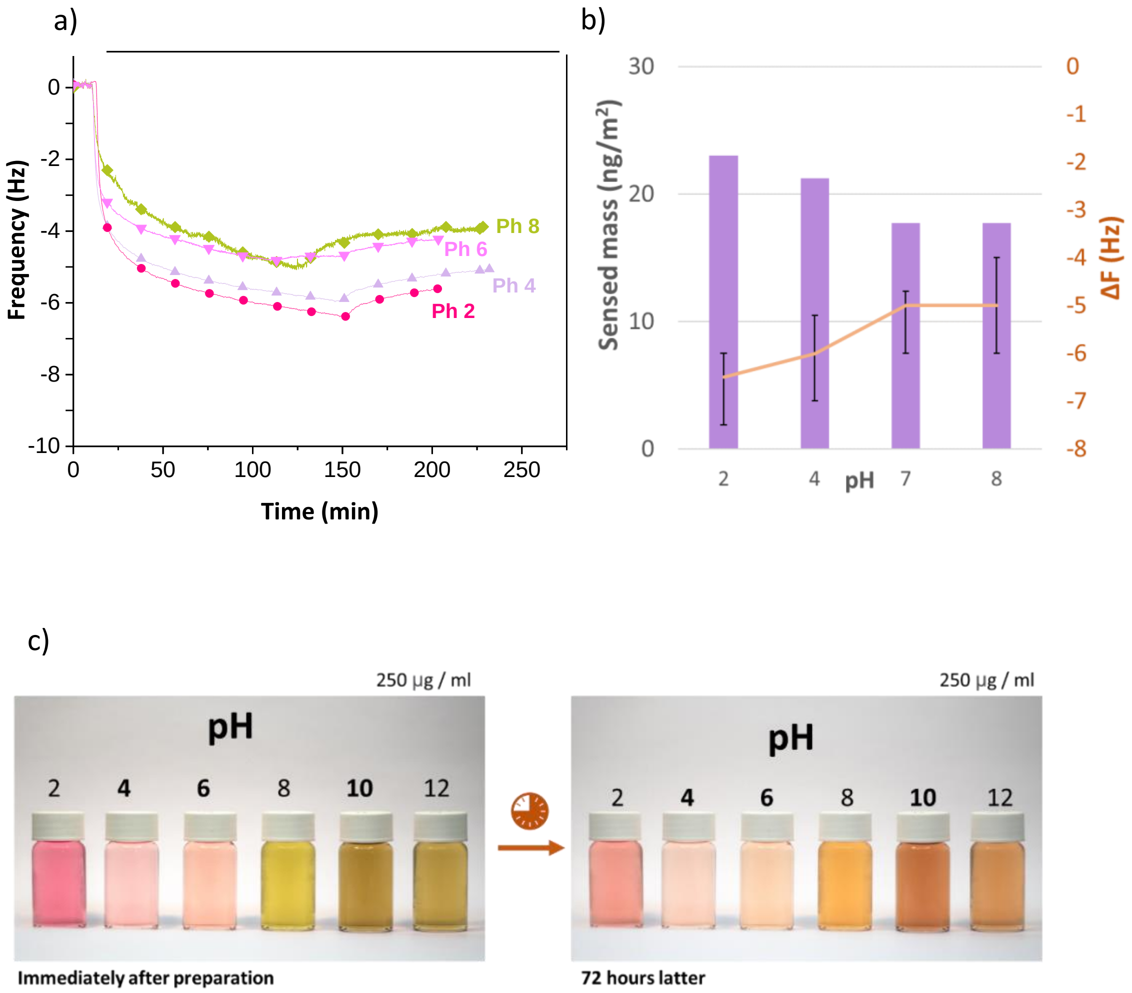

Figure S5. a) Change in oscillation frequency (fifth overtone) as a function of time of ROD in different pH conditions adsorbed onto CNF model films. b) Maximum sensed mass calculated from curve "a)" using Sauerbrey's relation c) visual appearance of ROD solutions (250 μg/mL) under different pH immediately after the solubilization and 72 h later.

Table S4. CIE L*, a*, b* color coordinates and visual colour, K/S values at minimum reflectance (Rmin) and at 480 and 520 nm, and light fastness (LF, evaluation 1-8 where 8 is the best value), washing fastness (evaluation 1-5, where 5 is the best value) and rubbing fastness (1-5) values of the dyed cotton fabrics. The total color change (CC) as ΔE values of the dyed samples before and after washing at pH 7 and pH 10 laundering liquors.

Sample	CIELab							CC CIELab		Washing fastness														Rubbing fastness	
No.	L*	a*	b*	K/S Rmin	K/S 480 nm	K/S 520 nm	LF	ΔE pH 7	ΔE pH 10	CC pH 7	CC pH 10	WO pH 7	WO pH 10	PAN pH 7	PAN pH 10	PES pH 7	PES pH 10	PA pH 7	PA pH 10	CO pH 7	CO pH 10	CA pH 7	CA pH 10	D(wf)	D(wp)
A1-0	70.14	3.88	13.72	9.13	0.54	0.51	5	10.13	7.25	3,Y,Str	3/4	3/4	4	4	5	5	5	4/5	5	4/5	5	4/5	5	4/5	4/5
A2-Al	57.39	-6.61	26.71	7.41	1.26	0.97	3	5.89	19.35	4	1/2	4	4	4/5	4/5	5	5	5	5	5	5	5	4/5	4/5	4/5
A3-Fe	37.46	-1.22	9.92	9.85	4.39	4.17	4	1.36	19.39	5	2	4/5	4/5	4	4	5	4	5	4	5	5	5	5	4	4
B1-Ch	60.29	11.70	6.06	6.26	1.00	1.17	1	25.31	17.13	2,G	1	4/5	4/5	4/5	4/5	4/5	4/5	4	4/5	3/4	4/5	4/5	4/5	4/5	4/5

Y=yellower, G=greener, Str=stronger, D(wf)=direction weft, D(wp)=direction warp

References

1 Müller-Maatsch J, Caligiani A, Tedeschi T, Elst K, Sforza S. Simple and Validated Quantitative 1 H NMR Method for the Determination of Methylation, Acetylation, and Feruloylation Degree of Pectin. *J Agric Food Chem*. 2014;62(37):9081-9087. doi:10.1021/jf502679s

2. Rakhshaee R, Panahandeh M. Stabilization of a magnetic nano-adsorbent by extracted pectin to remove methylene blue from aqueous solution: A comparative studying between two kinds of cross-likened pectin. *J Hazard Mater*. 2011;189(1-2):158-166. doi:10.1016/j.jhazmat.2011.02.013
